# Global incidence of spinal perineural Tarlov’s cysts and their morphological characteristics: a meta-analysis of 13,266 subjects

**DOI:** 10.1007/s00276-020-02644-y

**Published:** 2021-01-16

**Authors:** Tomasz Klepinowski, Wojciech Orbik, Leszek Sagan

**Affiliations:** Department of Neurosurgery, Pomeranian Medical University Hospital No. 1, Szczecin, Poland

**Keywords:** Tarlov cyst, Spinal perineural cyst, Meta-analysis, Incidence, Sacral cyst, Nerve root cyst

## Abstract

**Background:**

Spinal perineural Tarlov’s cysts (TCs) are considered incidental findings that occasionally might exert pressure upon nerve roots and correspond with patients’ signs and symptoms. Purpose of this meta-analysis is to deliver global incidence and characteristics (location, size, and shape) of TCs.

**Methods:**

Following PRISMA checklist, all major databases were searched by two authors for radiologic studies reporting incidence and morphologic features (location, size, and shape) of TCs. Anatomical Quality Assessment tool was applied for risk of bias evaluation. Meta-analysis of random-effects model was employed. Subgroup analysis for regional distribution, gender, sacral levels, age, correspondence with symptoms, and persistent genital arousal disorder (PGAD) were planned ahead.

**Results:**

22 radiologic studies of level 3 evidence involving 13,266 subjects were included. Global pooled prevalence of TCs was 4.18% (95% CI 2.47–6.30). Mean pooled sagittal diameter was 11.86 mm (95% CI 10.78–12.93). Sacral cysts strongly prevailed over the other segments. Of the sacral, S2 level was the most common (46.7% [95% CI 29.4–60.5]). Geographically, the highest incidence was found in Europe (6.07% [95% CI 1.49–13.00]), followed by North America (3.82% [95% CI 0.49–9.44]), and Asia (3.33% [95% CI 1.52–5.75]). TCs were more common in women than in men (5.84% vs 3.03%, *p* < 0.001, test of homogeneity, *χ*^2^). Subjects with PGAD had incidence of 37.87% (95% CI 2.45–81.75). TCs in pediatric population are rare—0.53% (95% CI 0.02–1.51). 15.59% of TCs corresponded with symptoms.

**Conclusions:**

Spinal perineural (Tarlov) cysts are found in a minority of population. S2 level of the sacral bone is affected most frequently. There is female predominance. Correspondence with symptoms is seen in less than one-fifth of TCs. Studies with stronger evidence level are needed to corroborate the results. The purported high incidence in PGAD requires confirmation in case–control studies for the risk-ratio calculation.

**Electronic supplementary material:**

The online version of this article (10.1007/s00276-020-02644-y) contains supplementary material, which is available to authorized users.

## Introduction

**Fig. 1:**
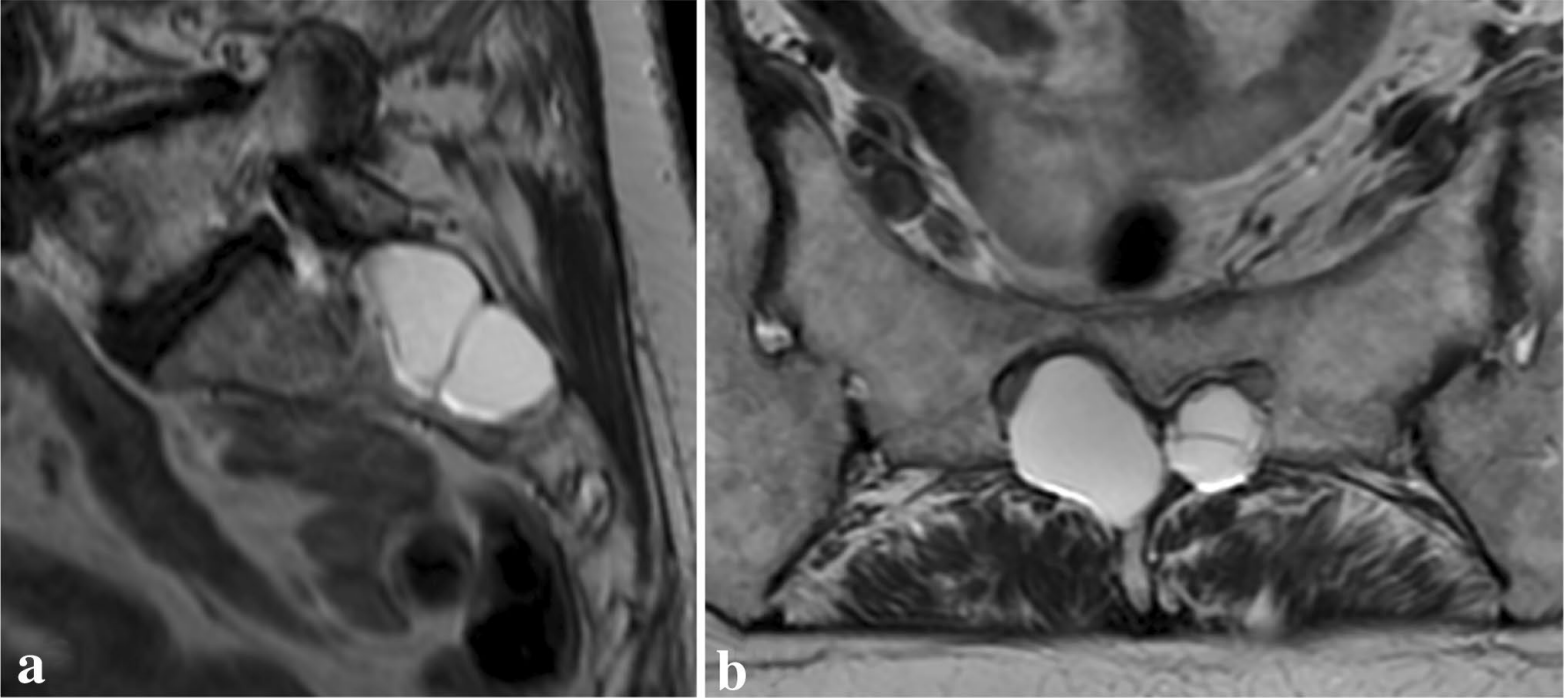
**a** Sagittal and **b** axial scans of magnetic resonance showing multiple Tarlov cysts at the sacral S2 level causing bone erosion

Spinal perineural Tarlov’s cysts are thought to be common radiological findings, often incidental and asymptomatic. They are a dilation of the nerve-root sheath and tend to communicate with subarachnoid space through a valve-like mechanism, which thus contain cerebrospinal fluid (CSF) as well as neural tissue [3, 6] (Fig. 1). Traditionally, according to Nabor’s classification (see Table 1), they are type 2 spinal meningeal cysts, albeit differentiation of these types might often be done only on histological inspection [16]. Incidence varies widely in the literature with reports of as high as 17.7% and as low as 0.38% [4, 14, 24]. Also, it has been a subject of controversy which spinal level and nerve root they are most often associated with. Although they are usually linked to the sacral segment of the spine, rarely, they may be seen at other levels, including the cervical and thoracic spine. Despite being mostly asymptomatic, they have been discussed as a potential cause of radiculopathy, persistent genital arousal disorder, as well as a source of CSF leak being a primary culprit of idiopathic intracranial hypotension [22]. Authors present the first meta-analysis aiming to discover global incidence and characteristics of Tarlov’s cysts.

**Table 1:** Nabor’s classification of spinal meningeal cysts [16]

Type	Description
I	Extradural cyst without nerve-root fibers
II	Extradural cyst with nerve-root fibers
III	Intradural cyst

## Methods

### Search strategy

Major electronic databases (EMBASE, SciELO, China National Knowledge Infrastructure, PubMed MEDLINE, Web of Science) were searched by two researchers (neurosurgical resident TK & WO) using a MeSH term ‘Tarlov cyst’ and corresponding synonyms. Full strategy is presented in Electronic Supplementary Material 1. The search started on the 1st August 2020 and was finalized by 6th August 2020. Year of publication was not restricted. Mendeley Desktop 1.19.4 was utilized for title importation, de-duplication, and creation of bibliographic details. Additionally, references of the included papers were checked for pertinent articles. PRISMA checklist is available as Electronic Supplementary Material 2.

### Eligibility

Analysis was limited to those studies that met our inclusion criteria: (1) clear incidence of Tarlov’s cysts among a specified group of subjects; (2) the incidence regarded the group of at least 10 subjects. Papers were excluded if any criterion of the following was found: (1) conference abstracts, (2) commentaries, (3) letters to the Editors, (4) insufficient data, (5) incidence of Tarlov’s cysts was aggregated with other lesions and could not be disaggregated, (6) case reports, and (7) series with less than 10 subjects among whom presence of the cysts was evaluated. TK and WO are fluent in English and Polish. In case of other language, professional translators were inquired.

### Data extraction

Data were extracted by two of us (TK and WO). The following characteristics were of interest: (1) total number of subjects evaluated for presence of Tarlov’s cysts, (2) number of individuals with at least one TC, (3) number of individuals with multiple TCs, (4) total number of TCs in the group, (5) number of patients with only one, only two, only three (and so on) TCs, respectively, (6) number of TCs in sacral, lumbar, thoracic, or cervical region, respectively, (7) number of TCs corresponding with symptoms (excluding back pain) or signs, (8) number of TCs not corresponding with symptoms or signs, (9) number of TCs in patients with persistent genital arousal disorder, (10) number of TCs in patients with transitional lumbosacral vertebra, (11) mean age of the group with TCs, (12) number of adults and children with TCs, (13) size of TCs in millimeters (the maximal axial diameter), (14) prevalence of TCs with respect to nerve-root level, (15) prevalence with regard to sex, (16) shape of TCs, (17) region of origin, and (18) modality of imaging. Data were collected only if it could have been disaggregated. In several cases, the disaggregated data were provided by the corresponding authors who were communicated with.

### Quality and risk of bias assessment

Two researchers are TK and WO. Risk of bias was assessed in two ways: quantitatively and qualitatively. Qualitative analysis was performed by means of AQUA tool (Anatomical Quality Assessment) [8] independently by two reviewers. In case of disagreement, a senior neurosurgeon (LS) was reached for consensus. AQUA tool appreciates quality of the study in five domains: (1) objectives and subject characterization, (2) study design, (3) methodology, (4) descriptive anatomy, and (5) reporting results. Each domain asks a series of binary signaling questions. If a single signaling question within a domain was answered ‘No’, then consensus was reached whether the entire domain should be ‘High risk’ or ‘Low risk’. On the other hand, in case of two or more signaling questions being answered ‘No’, then the domain was instantly marked as constituting a ‘High risk’. On the other hand, quantitative analysis of the risk of bias was conducted by delineating a mathematically grounded funnel plot and evaluating its symmetry.

### Statistics

Formal statistical estimation of pooled prevalence was executed using software MetaXL 5.3, EpiGear International Pty Ltd. (Brisbane, Australia). Estimation of mean sagittal diameter was executed with Comprehensive Meta-Analysis V3 (Englewood, New Jersey, USA). For single-categorical variables, pooled prevalence was estimated with corresponding confidence intervals of 95%. *I*^2^ and *χ*^2^ were incorporated into evaluation of heterogeneity. *I*^2^ value was interpreted with classic approach: 0–40%: not be important, 30–60% moderate heterogeneity, 50–90%: substantial heterogeneity, and 75–100%: considerable heterogeneity. Arbitrary p value of < 10% was set for Cochrane *Q* significance (< 0.10). Conversely, significance of comparative tests was agreed to standard < 5% (< 0.05). Random-effects model for meta-analysis of pooled prevalence was applied. Subgroup differences in prevalence were compared using test of homogeneity based on *χ*^2^ (Statistica 13.3.0, TIBCO Software Inc, Palo Alto, USA).

### Subgroup analysis

Subgroup analysis was planned with regard to geographical regions, specific entities such as persistent genital arousal disorder, sex, and age (children and adults). Distribution within the sacral spine was also analyzed to determine which sacral level is affected most commonly.

## Results

### Selection process

**Fig. 2:**
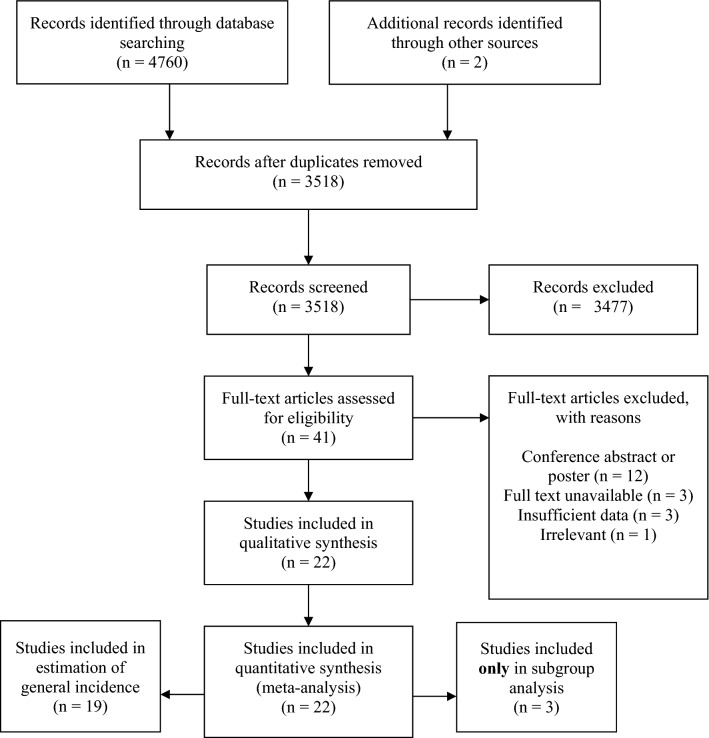
Process of study selection

Initial search yielded 4762 records including 2 from references of the pertinent articles. Following the de-duplication, 3518 titles and abstracts were checked for relevance to the topic. 41 full-text articles were assessed for eligibility (Fig. 2). In total, 22 papers were included in meta-analysis [2, 4, 5, 9–15, 17–21, 23–29]: 19 for estimation of the general incidence as well as for subgroup analysis, whereas three articles were included solely in subgroup analysis without involving them in the general pool, because they pertained specifically to small groups of patients with symptoms of PGAD, two of them presented unusually high incidence of TC, and hence were deemed to pose a risk of bias for the general pool.

### Risk of bias and quality

**Fig. 3:**
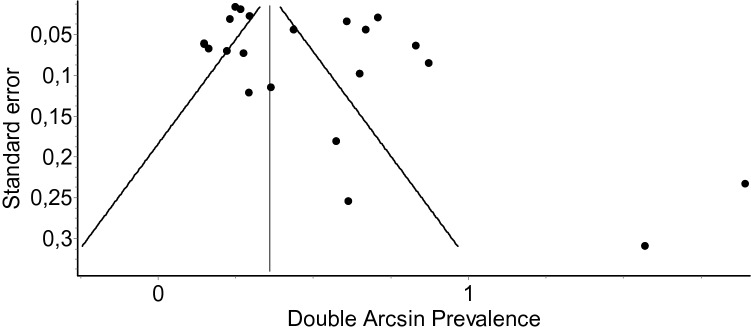
Funnel plot quantitatively illustrating the risk of bias of Tarlov cyst prevalence in the general pool

Tabular display of AQUA tool summary is presented in Table 2. The highest risk was related with methodology, whereas the lowest risk was attributed to study design and reporting results. The funnel plot is presented in Fig. 3.

**Table 2:** Summary of risk of bias evaluation by means of an Anatomical Quality Assessment (AQUA) tool

References	Risk of Bias				
	Objectives and study characteristics	Study design	Methodology	Descriptive anatomy	Reporting of results
Burdan et al. [2]	Low	Low	Low	Low	High
Gleeson et al. [4]	Low	Low	Low	High	Low
Gopalakrishnan et al. [5]	High	Low	High	High	Low
Jeong et al. [9]	Low	Low	Low	High	Low
Joo et al. [10]	Low	Low	High	High	Low
Komisaruk and Lee [11]	High	High	High	Low	Low
Kuhn et al. [12]	Low	Low	High	Low	Low
Langdown et al. [13]	Low	Low	High	Low	Low
Larsen et al. [14]	High	High	High	Low	Low
Lim et al. [15]	Low	Low	High	Low	Low
Oaklander et al. [17]	High	High	High	High	High
Park et al. [18]	Low	Low	Low	Low	Low
Paulsen et al. [19]	High	Low	High	High	High
Petrasic et al. [20]	Low	Low	High	High	Low
Pink et al. [21]	High	High	High	High	High
Ramadorai et al. [23]	Low	Low	High	High	Low
Ramirez et al. [24]	Low	High	High	High	Low
Senoglu et al. [25]	Low	Low	High	Low	Low
Shi et al. [26]	High	Low	High	High	Low
Tani et al. [27]	Low	Low	High	High	Low
Zacharakis et al. [28]	Low	Low	High	High	Low
Zeitoun et al. [29]	Low	Low	High	High	Low

### Characteristics of the included studies

Characteristics are presented in Table 3. In total, 13,266 subjects were included into the meta-analysis. 21 studies sought Tarlov’s cysts in MRI scans, whereas one study evaluated only myelograms. No study with evidence stronger than level 3 was identified. Time frame of the studies spanned from 1980 to 2020. Age was known in 14 studies (*n* = 6717). Thus, mean age of those in the main cohort was 48.9 years. Geographical distribution was as follows: 8 studies from North America (*n* = 1298), 6 from Europe (*n* = 5903), 7 from Asia (*n* = 5808), 1 from Africa (*n* = 217), and neither from Australia nor South America.

**Table 3:** Characteristics of the studies

References	Global region	Imaging	Subjects	Tarlov cyst incidence (%)
Burdan et al. [2]	Europe	MRI	842	8.91
Gleeson et al. [4]	Europe	MRI	260	0.38
Gopalakrishnan et al. [5]	Asia	MRI	200	1.00
Jeong et al. [9]	Asia	MRI	494	10.73
Joo et al. [10]	Asia	MRI	2669	1.72
Komisaruk and Lee [11]*	North America	MRI	18	66.67
Kuhn et al. [12]	Europe	MRI	1100	12.00
Langdown et al. [13]	Europe	MRI	3535	1.53
Larsen et al. [14]	Europe	Myelography	136	17.65
Lim et al. [15]	North America	MRI	242	16.12
Oaklander et al. [17]*	North America	MRI	10	50.00
Park et al. [18]	Asia	MRI	1268	2.13
Paulsen et al. [19]	North America	MRI	500	4.60
Petrasic et al. [20]	North America	MRI	185	1.62
Pink et al. [21]*	North America	MRI	15	6.67
Ramadorai et al. [23]	North America	MRI	67	1.49
Ramirez et al. [24]	North America	MRI	261	0.38
Senoglu et al. [25]	Asia	MRI	1000	1.30
Shi et al. [26]	Asia	MRI	75	2.67
Tani et al. [27]	Asia	MRI	102	9.80
Zacharakis et al. [28]	Europe	MRI	30	6.67
Zeitoun et al. [29]	Asia	MRI	217	0.46

Excluded from estimation of the general pooled prevalence, included only in subgroup analysis

*Patients with persistent genital arousal disorder

### Pooled estimates and subgroup analysis

**Fig. 4:**
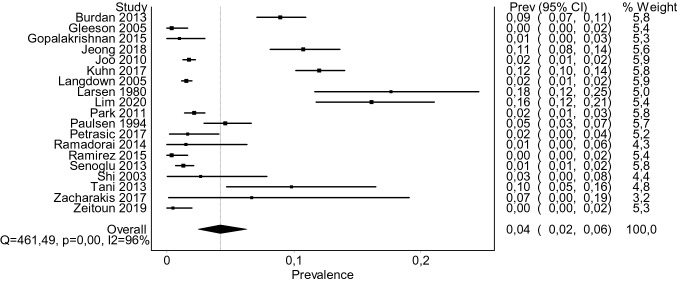
A forest plot depicting global pooled prevalence of Tarlov cysts. Overall prevalence was estimated at 4.18% (95% CI 2.47–6.30)

Random-effects model estimated global pooled prevalence of Tarlov’s cysts to be 4.18% (95% CI 2.47–6.30, *Q* = 461.49, *p* < 0.01, *I*^2^ = 96.1%; see Fig. 4). Considering geographical distribution, the highest incidence of TCs was noted in Europe (6.07% [95% CI 1.49–13.00], *Q* = 269.62, *p* < 0.001, *I*^2^ = 98.15%), followed by North America (3.82% [95% CI 0.49–9.44], *Q* = 65.62, *p* < 0.001, *I*^2^ = 92.4%), Asia (3.33% [95% CI 1.52–5.75], *Q* = 89.32, *I*^2^ = 93.28%, *p* < 0.001), and Africa (0.67% [95% CI 0.00–1.97]; a single study, Cochran’s *Q* was not calculated). Comparing all four regions, the geographical differences were not significant (*p* = 0.213; test of homogeneity, Chi squared). However, direct comparison revealed that incidence in Europe was significantly higher than in Asia (*p* < 0.01) or in North America (*p* < 0.01). Incidence in Asia was not dissimilar from that in North America (*p* = 0.607). Subjects with persistent genital arousal disorder showed prevalence of 37.87% (95% CI 2.45–81.75; *Q* = 14.33, *I*^2^ = 86.0%). Size of the cysts was measured in three studies (*n* = 1960). Mean pooled diameter was 11.86 mm (95% CI 10.78–12.93, variance = 0.302, *Z* value = 21.59, *p* < 0.01, *I*^2^ = 83.5%). Incidence in women was 7.01% (95% CI 2.57–13.21, *Q* = 221.07, *p* < 0.01, *I*^2^ = 96.38%), whereas in men 4.05% (95% CI 1.07–8.57, *Q* = 27.86, *p* < 0.01, *I*^2^ = 89.23%). Tarlov’s cysts in pediatrics were rare (0.53% [95% CI 0.02–1.51, *Q* = 1.61, *p* = 0.448, *I*^2^ = 0%]). Shape was oval/rounded in 58.3% of TCs and tubular in 41.7% (*Q* = 30.13, *p* < 0.01, *I*^2^ = 96.7%). Of the cysts that were found, 57.4% were single, whereas in 42.6%, multiple TCs were seen (*Q* = 30.21, *p* < 0.01, *I*^2^ = 76.8). Only 15.59% of TCs corresponded with symptoms of neural compromise (radiculopathy, chronic cauda equina syndrome, myelopathy, PGAD, or the like) (see Table 4).

**Table 4:** Subgroup analysis of Tarlov cyst prevalence

Subgroup	Number of studies (subjects)	Pooled prevalence (95% CI)	*I*^2^ heterogeneity (%)	*p* value of Cochran *Q*
Age group				
Adults	13 (11965)	5.51% (3.11–8.53)	97.1	< 0.001
Pediatrics	3 (428)	0.53% (0.02–1.51)	0	0.448
Persistent genital arousal disorder	3 (43)	37.87% (2.45–81.75)	86.0	0.001
Sacral levels*	9 (4274)		85.7	< 0.001
S1		21.7% (9.3–35.0)		
S2		46.7% (29.4–60.5)		
S3		26.4% (12.8–40.2)		
S4		5.2% (0.0–12.9)		
Gender	9 (4333)			
Women	9 (3103)	7.01% (2.57–13.21)	96.4	< 0.001
Men	4 (1230)	4.05% (1.07–8.57)	91.75	< 0.001
Geographical distribution				
North America	8 (1298)	3.82% (0.49–9.44)	92.4	< 0.001
Europe	6 (5903)	6.07% (1.49–13.00)	98.15	< 0.001
Asia	7 (5808)	3.33% (1.52–5.75)	93.3	< 0.001
Africa	1 (217)	0.67% (0.00–1.97)	0	–
Shape of TCs	2 (1236)		96.7	< 0.001
oval/rounded		58.3% (8–100)		
Tubular		41.7% (0–92)		
Patients with	8 (1893)		76.8	< 0.001
Only one TC		57.4% (34.9–78.5)		
Multiple TCs		42.6% (21.5–65.1)		
TCs corresponding with symptoms	8 (2633)	15.59% (2.47–35.24)	77.9	< 0.001

*S1–S4 prevalence rates were calculated to determine the most commonly affected sacral level

## Discussion

Although in literature a varyingly wide range of incidence of TCs is reported, the present meta-analysis shows that mean global pooled prevalence is 4.27% and the confidence interval is relatively narrow (95% CI 2.56–6.38). The highest general incidence—17.65%—was suggested by Larsen et al. in 1980 [14]. As contributory and impactful their research might have been, it was the oldest of the included studies and also the only one that took advantage of myelography. All the other papers were from the new millennium and evaluated cysts using magnetic resonance imaging. This crucial methodological difference might partially explain the discrepancy.

8 studies addressed gender-specific prevalence. In women, TCs occurred at 7.01% (95% CI 2.57–13.21) whereas men had prevalence of 4.05% (95% CI 1.07–8.57). Apparently, females are more often affected by TCs (*p* = 0.0003, test of homogeneity, *χ*^2^). Literature has not explored the causes of this sex-related predominance, yet. Our hypothesis is that formation of TCs in adult females might be associated with the level of primary female sex hormones, since in pediatrics, when these hormones are usually low, TCs are an exceptional finding [12]. This hypothesis might be corroborated in future by conducting case–control research studies comparing a group of hypogonadism with healthy controls.

### Correspondence with symptoms

This meta-analysis shows that only about 15.59% of TCs are symptomatic. This is somewhat close to what has been suggested in the past by Paulsen et al. who indicated that approximately only 1 in 5 TCs might produce clinically significant symptoms [19]. Clinical picture that may truly be related to the presence of TCs stems from neural compromise and most commonly consists of radicular pain in the relevant dermatomal distribution or motor weakness, rarely myelopathy if a TC is located above the conus medullaris [7, 13, 19]. Persistent genital arousal disorder is under debate as an entity that could be linked with TCs [11]. Non-specific back pain was not attributed to TCs in the present meta-analysis.

### Sacral segments

Sacral perineural cysts were the ones to be documented most consistently. It was a subject of controversy which sacral level is usually involved. Incidence of S2 TCs was declared highest in three studies [2, 9, 15]. S3 was the most prevalent in two papers, whereas S1 in one [12, 14, 25]. Pooling the data revealed that, in fact, S2 level is the one to be disturbed most frequently (46.7% [95% CI 29.4–60.5], *Q* = 49.12, *I*^2^ = 85.7%, *p* < 0.001).

### Tarlov cysts and persistent genital arousal disorder

Subgroup analysis reveals that patients with PGAD, mostly women, might be more prone to developing TCs. Two of three studies involving those patients gave an account of high TC incidence (50–66.67%) [1, 17]. However, one study stated to the contrary (6.67%), indicating that the previously mentioned studies might have been biased [21]. Pooled prevalence for the time being is 37.87% Currently, more studies of larger samples, optimally of case–control design, would be appreciated to dispel doubts.

### Implications, limitations, and future directions

Despite being comprehensive, this meta-analysis is not free from limitations. High methodological bias found in many papers is highlighted, primarily due to the fact that in many of them, measures were not taken to reduce intra-/interobserver variability. Small number of studies about PGAD is the reason of the wide confidence interval of incidence in this group. It, however, indicates that PGAD might be associated with TCs. Therefore, case–control studies would be of value for calculation of the risk ratio. Based on the findings of this study, it is suggested that more studies on incidence of TCs be conducted in South America, Africa, and Australia. Moreover, results of this meta-analysis and the emphasized gaps in knowledge can be used in future research on Tarlov’s cysts. As all but one of the studies assessed only lumbosacral spine MRI and not whole-spine MRI, there exists some risk of bias regarding counting the vertebrae in individuals with transitional abnormalities. Since only a handful of papers delineated morphological features of the cysts such as size or shape, as for now, it is difficult to seek link between these characteristics and clinical picture. Especially, it is still problematic to determine quantitative relation between TCs and the most frequent complaints expressed by patients, which is radicular pain and local back pain. Moreover, there are not enough data to univocally answer the question whether spinal cord or cauda equina might herniate into the TCs. Hence, more precise depiction is encouraged in upcoming studies.

## Conclusion

Spinal perineural Tarlov’s cysts are found in a minority of population. Women tend to develop TCs more often than men. Perineural cysts in pediatrics seem to be rare findings. Incidence in Europe appears to be higher than in other continents. S2 level of the sacral bone is affected most frequently. Possibly high incidence in PGAD requires confirmation in case–control studies for the risk-ratio calculation. Studies with evidence stronger than level 3 are needed to corroborate the results of this meta-analysis.

## Supporting information

Below is the link to the electronic supplementary material. Supplementary material 1 (DOCX 13 kb) Supplementary material 2 (DOC 67 kb)
