# Supplementary material for: Global incidence of spinal perineural Tarlov’s cysts and their morphological characteristics: a meta-analysis of 13,266 subjects
Source: Surg Radiol Anat. 2021 Jan 16;43(6):855–63. doi: 10.1007/s00276-020-02644-y (PMC8164596; doi:10.1007/s00276-020-02644-y)
Supplement: Supplementary file 1 — Supplementary material 1 (DOCX 13 kb) [file 276_2020_2644_MOESM1_ESM.docx]

**Electronic Supplementary Material No. 1**

This material is part of the manuscript entitled ‘Global incidence of spinal perineural Tarlov’s cysts and their morphological characteristics: a meta-analysis of 13,266 subjects’ authored by T. Klepinowski, W. Orbik, L. Sagan.

Corresponding author: T. Klepinowski

Affiliation: Department of Neurosurgery, Pomeranian Medical University Hospital No 1, Szczecin, Poland

E-mail address: [tomasz.klepinowski@pum.edu.pl](mailto:tomasz.klepinowski@pum.edu.pl)

((((((((((((("tarlov cysts"[MeSH Terms] OR ("tarlov"[All Fields] AND "cysts"[All Fields])) OR "tarlov cysts"[All Fields]) OR ("tarlov"[All Fields] AND "cyst"[All Fields])) OR "tarlov cyst"[All Fields]) OR ((((("spinal"[All Fields] OR "spinalization"[All Fields]) OR "spinalized"[All Fields]) OR "spinally"[All Fields]) OR "spinals"[All Fields]) AND (((("tarlov cysts"[MeSH Terms] OR ("tarlov"[All Fields] AND "cysts"[All Fields])) OR "tarlov cysts"[All Fields]) OR ("perineural"[All Fields] AND "cyst"[All Fields])) OR "perineural cyst"[All Fields]))) OR ((((("spinal nerve roots"[MeSH Terms] OR (("spinal"[All Fields] AND "nerve"[All Fields]) AND "roots"[All Fields])) OR "spinal nerve roots"[All Fields]) OR (("spinal"[All Fields] AND "nerve"[All Fields]) AND "root"[All Fields])) OR "spinal nerve root"[All Fields]) AND ((((((((((((((((("cysts"[MeSH Terms] OR "cysts"[All Fields]) OR "cyst"[All Fields]) OR "neurofibroma"[MeSH Terms]) OR "neurofibroma"[All Fields]) OR "neurofibromas"[All Fields]) OR "tumor s"[All Fields]) OR "tumoral"[All Fields]) OR "tumorous"[All Fields]) OR "tumour"[All Fields]) OR "neoplasms"[MeSH Terms]) OR "neoplasms"[All Fields]) OR "tumor"[All Fields]) OR "tumour s"[All Fields]) OR "tumoural"[All Fields]) OR "tumourous"[All Fields]) OR "tumours"[All Fields]) OR "tumors"[All Fields]))) OR (((((((((("meningeal"[All Fields] OR "meninges"[MeSH Terms]) OR "meninges"[All Fields]) OR "meninge"[All Fields]) OR "meningism"[MeSH Terms]) OR "meningism"[All Fields]) OR "meningisms"[All Fields]) OR "meningitis"[MeSH Terms]) OR "meningitis"[All Fields]) OR "meningitides"[All Fields]) AND ((((((((((((((((("cysts"[MeSH Terms] OR "cysts"[All Fields]) OR "cyst"[All Fields]) OR "neurofibroma"[MeSH Terms]) OR "neurofibroma"[All Fields]) OR "neurofibromas"[All Fields]) OR "tumor s"[All Fields]) OR "tumoral"[All Fields]) OR "tumorous"[All Fields]) OR "tumour"[All Fields]) OR "neoplasms"[MeSH Terms]) OR "neoplasms"[All Fields]) OR "tumor"[All Fields]) OR "tumour s"[All Fields]) OR "tumoural"[All Fields]) OR "tumourous"[All Fields]) OR "tumours"[All Fields]) OR "tumors"[All Fields]) AND ((("spine"[MeSH Terms] OR "spine"[All Fields]) OR "spines"[All Fields]) OR "spine s"[All Fields]))) OR (("extra"[All Fields] OR "extras"[All Fields]) AND ((((((((("meningeal"[All Fields] OR "meninges"[MeSH Terms]) OR "meninges"[All Fields]) OR "meninge"[All Fields]) OR "meningism"[MeSH Terms]) OR "meningism"[All Fields]) OR "meningisms"[All Fields]) OR "meningitis"[MeSH Terms]) OR "meningitis"[All Fields]) OR "meningitides"[All Fields]) AND ((((((((((((((((("cysts"[MeSH Terms] OR "cysts"[All Fields]) OR "cyst"[All Fields]) OR "neurofibroma"[MeSH Terms]) OR "neurofibroma"[All Fields]) OR "neurofibromas"[All Fields]) OR "tumor s"[All Fields]) OR "tumoral"[All Fields]) OR "tumorous"[All Fields]) OR "tumour"[All Fields]) OR "neoplasms"[MeSH Terms]) OR "neoplasms"[All Fields]) OR "tumor"[All Fields]) OR "tumour s"[All Fields]) OR "tumoural"[All Fields]) OR "tumourous"[All Fields]) OR "tumours"[All Fields]) OR "tumors"[All Fields]) AND (((("spinal nerve roots"[MeSH Terms] OR (("spinal"[All Fields] AND "nerve"[All Fields]) AND "roots"[All Fields])) OR "spinal nerve roots"[All Fields]) OR (("spinal"[All Fields] AND "nerve"[All Fields]) AND "root"[All Fields])) OR "spinal nerve root"[All Fields]) AND ((((((((("dietary fiber"[MeSH Terms] OR ("dietary"[All Fields] AND "fiber"[All Fields])) OR "dietary fiber"[All Fields]) OR "fiber"[All Fields]) OR "fibre"[All Fields]) OR "fiber s"[All Fields]) OR "fiberized"[All Fields]) OR "fibers"[All Fields]) OR "fibre s"[All Fields]) OR "fibres"[All Fields]))) OR ((((("spinal"[All Fields] OR "spinalization"[All Fields]) OR "spinalized"[All Fields]) OR "spinally"[All Fields]) OR "spinals"[All Fields]) AND ("extradural"[All Fields] OR "extradurally"[All Fields]) AND ((((((("arachnoid"[MeSH Terms] OR "arachnoid"[All Fields]) OR "arachnoids"[All Fields]) OR "arachnoidal"[All Fields]) OR "arachnoideal"[All Fields]) OR "arachnoiditis"[MeSH Terms]) OR "arachnoiditis"[All Fields]) OR "arachnoiditides"[All Fields]) AND ((((("pouch"[All Fields] OR "pouch s"[All Fields]) OR "pouche"[All Fields]) OR "pouches"[All Fields]) OR "pouching"[All Fields]) OR "pouchs"[All Fields]))) OR ((((("tarlov cysts"[MeSH Terms] OR ("tarlov"[All Fields] AND "cysts"[All Fields])) OR "tarlov cysts"[All Fields]) OR (("cyst"[All Fields] AND "nerve"[All Fields]) AND "root"[All Fields])) OR "cyst of the nerve root"[All Fields]) AND (((((("foreskin"[MeSH Terms] OR "foreskin"[All Fields]) OR "sheath"[All Fields]) OR "sheathed"[All Fields]) OR "sheathes"[All Fields]) OR "sheathing"[All Fields]) OR "sheaths"[All Fields]))) OR ((((("diverticulae"[All Fields] OR "diverticulas"[All Fields]) OR "diverticulum"[MeSH Terms]) OR "diverticulum"[All Fields]) OR "diverticula"[All Fields]) AND ((((((((("meningeal"[All Fields] OR "meninges"[MeSH Terms]) OR "meninges"[All Fields]) OR "meninge"[All Fields]) OR "meningism"[MeSH Terms]) OR "meningism"[All Fields]) OR "meningisms"[All Fields]) OR "meningitis"[MeSH Terms]) OR "meningitis"[All Fields]) OR "meningitides"[All Fields]) AND "sac"[All Fields])) OR ((((("diverticulae"[All Fields] OR "diverticulas"[All Fields]) OR "diverticulum"[MeSH Terms]) OR "diverticulum"[All Fields]) OR "diverticula"[All Fields]) AND ((("nerve"[All Fields] OR "nerve s"[All Fields]) OR "nerved"[All Fields]) OR "nerves"[All Fields]) AND (((((("foreskin"[MeSH Terms] OR "foreskin"[All Fields]) OR "sheath"[All Fields]) OR "sheathed"[All Fields]) OR "sheathes"[All Fields]) OR "sheathing"[All Fields]) OR "sheaths"[All Fields]))) OR ((((("spinal"[All Fields] OR "spinalization"[All Fields]) OR "spinalized"[All Fields]) OR "spinally"[All Fields]) OR "spinals"[All Fields]) AND (((("arachnoid cysts"[MeSH Terms] OR ("arachnoid"[All Fields] AND "cysts"[All Fields])) OR "arachnoid cysts"[All Fields]) OR ("arachnoid"[All Fields] AND "cyst"[All Fields])) OR "arachnoid cyst"[All Fields]))) NOT (((((((((((((((((("sheep"[MeSH Terms] OR "sheep"[All Fields]) OR "sheeps"[All Fields]) OR "sheep s"[All Fields]) OR "sheep, domestic"[MeSH Terms]) OR ("sheep"[All Fields] AND "domestic"[All Fields])) OR "domestic sheep"[All Fields]) OR (("dogs"[MeSH Terms] OR "dogs"[All Fields]) OR "dog"[All Fields])) OR (("rats"[MeSH Terms] OR "rats"[All Fields]) OR "rat"[All Fields])) OR ((("rabbit s"[All Fields] OR "rabbits"[MeSH Terms]) OR "rabbits"[All Fields]) OR "rabbit"[All Fields])) OR ((((((("experimental"[All Fields] OR "experimentally"[All Fields]) OR "experimentals"[All Fields]) OR "experimentation"[All Fields]) OR "experimentations"[All Fields]) OR "experimenter"[All Fields]) OR "experimenter s"[All Fields]) OR "experimenters"[All Fields])) OR ((((((((((((((((("model"[All Fields] OR "model s"[All Fields]) OR "modeled"[All Fields]) OR "modeler"[All Fields]) OR "modeler s"[All Fields]) OR "modelers"[All Fields]) OR "modeling"[All Fields]) OR "modelings"[All Fields]) OR "modelization"[All Fields]) OR "modelizations"[All Fields]) OR "modelize"[All Fields]) OR "modelized"[All Fields]) OR "modelled"[All Fields]) OR "modeller"[All Fields]) OR "modellers"[All Fields]) OR "modelling"[All Fields]) OR "modellings"[All Fields]) OR "models"[All Fields])) OR "neoplasm"[All Fields]) OR "neurofibroma"[All Fields]) OR (("neurofibromatoses"[MeSH Terms] OR "neurofibromatoses"[All Fields]) OR "neurofibromatosis"[All Fields])) OR "tumour"[All Fields]) OR "tumor"[All Fields]) OR "tumorous"[All Fields]) OR "tumoural"[All Fields])
